# A Machine Learning Approach for Recommending Herbal Formulae with Enhanced Interpretability and Applicability

**DOI:** 10.3390/biom12111604

**Published:** 2022-10-31

**Authors:** Won-Yung Lee, Youngseop Lee, Siwoo Lee, Young Woo Kim, Ji-Hwan Kim

**Affiliations:** 1 College of Korean Medicine, Dongguk University, 32 Dongguk-ro, Ilsandong-gu, Goyang-si 10326, Korea; 2 Korean Medicine Data Division, Korean Institute of Oriental Medicine, 1672 Yuseong-daero, Daejeon 34054, Korea; 3 College of Korean Medicine, Gachon University, Seongnam 13120, Korea

**Keywords:** herbal formula, Korean medicine, recommendation model, LIME

## Abstract

Herbal formulae (HFs) are representative interventions in Korean medicine (KM) for the prevention and treatment of various diseases. Here, we proposed a machine learning-based approach for HF recommendation with enhanced interpretability and applicability. A dataset consisting of clinical symptoms, Sasang constitution (SC) types, and prescribed HFs was derived from a multicenter study. Case studies published over 10 years were collected and curated by experts. Various classifiers, oversampling methods, and data imputation techniques were comprehensively considered. The local interpretable model-agnostic explanation (LIME) technique was applied to identify the clinical symptoms that led to the recommendation of specific HFs. We found that the cascaded deep forest (CDF) model with data imputation and oversampling yielded the best performance on the training set and holdout test set. Our model also achieved top-1 and top-3 accuracies of 0.35 and 0.89, respectively, on case study datasets in which clinical symptoms were only partially recorded. We performed an expert evaluation on the reliability of interpretation results using case studies and achieved a score close to normal. Taken together, our model will contribute to the modernization of KM and the identification of an HF selection process through the development of a practically useful HF recommendation model.

## 1. Introduction

An herbal formula (HF) is a representative intervention in Korean medicine (KM) [1]. KM doctors select and administer HFs consisting of medical herbs to patients according to their clinical diagnoses. HFs play a crucial role in the prevention and treatment of various diseases. For instance, a nationwide multicenter study suggested that Chunggan granules, a type of HF, effectively protect against liver fibrosis in patients with chronic liver diseases [2]. Additionally, a double-blind clinical trial indicated that Gyejigachulbutang (Gui-Zhi-Jia-Shu-Fu-Tang for China and Keishikajutsubuto for Japan) might be effective for non-obese patients with degenerative knee osteoarthritis [3]. During the COVID-19 pandemic, a systematic review and meta-analysis showed that HFs may provide potential benefits to patients suffering from COVID-19 [4].

Despite recent technological advances, the core process of diagnosing and prescribing HFs still mainly depends on the experience of individual KM doctors. Generally, KM doctors comprehensively identify the pathophysiological patterns (also called “zheng”) of patients based on given clusters of symptoms and signs through observation, listening, asking, and cutting [5]. By comprehensively considering the identified patterns, the KM doctors administer HFs to ameliorate the major complaints of the patients. Although this procedure provides a comprehensive treatment strategy for various diseases, its usefulness is limited by the high variability among experts and its reliance on implicit knowledge. The limitations of this diagnostic process pose a formidable challenge to the standardization and modernization of KM. Therefore, it is necessary to develop an interpretable and practically useful model that can explicitly understand the HF recommendation process.

The conventional approaches used for HF recommendation systems mainly rely on expert knowledge. The majority of these conventional approaches are clinical practice guidelines that collect diagnostic criteria and determine administrable HFs based on expert consensus or questionnaires that can classify pathological patterns or formula indications [6,7,8]. These approaches can act as indirect guides in clinical practice, but they cannot directly assist in the process of HF selection for individual patients. Researchers have thus attempted to develop data-driven models based on machine learning approaches. Seq2seq models, probabilistic graphic models, and multistep analysis methods are representative machine learning models that have been employed to develop HF recommendation systems [9,10,11]. In particular, Zhou et al. developed an HF recommendation model by combining phenotype and molecular information based on a convolutional neural network and a network embedding model [12]. Although these approaches can be useful for recommending prescriptions to patients, the applicability and/or interpretability of their predictive results remains an unresolved issue.

Interpretable machine learning methods have been proposed and have been applied to various fields, including the biomedical field. The local interpretable model-agnostic explanation (LIME) technique proposed by Ribeiro et al. is a representative model-agnostic interpretation method [13]. The characteristic of LIME is that by observing how the output is affected by a perturbed input, it can be applied to any supervised learning model to provide an interpretation of the given instance. Furthermore, incomplete data are an unavoidable problem and one that should be addressed during the data preprocessing stage. Missing values in the input dataset can cause the performance of the utilized classification algorithm to decrease, so data imputation algorithms have been proposed to solve this problem. Data imputation can also be applied to datasets containing nan values, increasing the applicability of trained models. Therefore, combining data imputation and LIME would be a useful approach for increasing the applicability and interpretability of prediction results.

**Figure 1:**
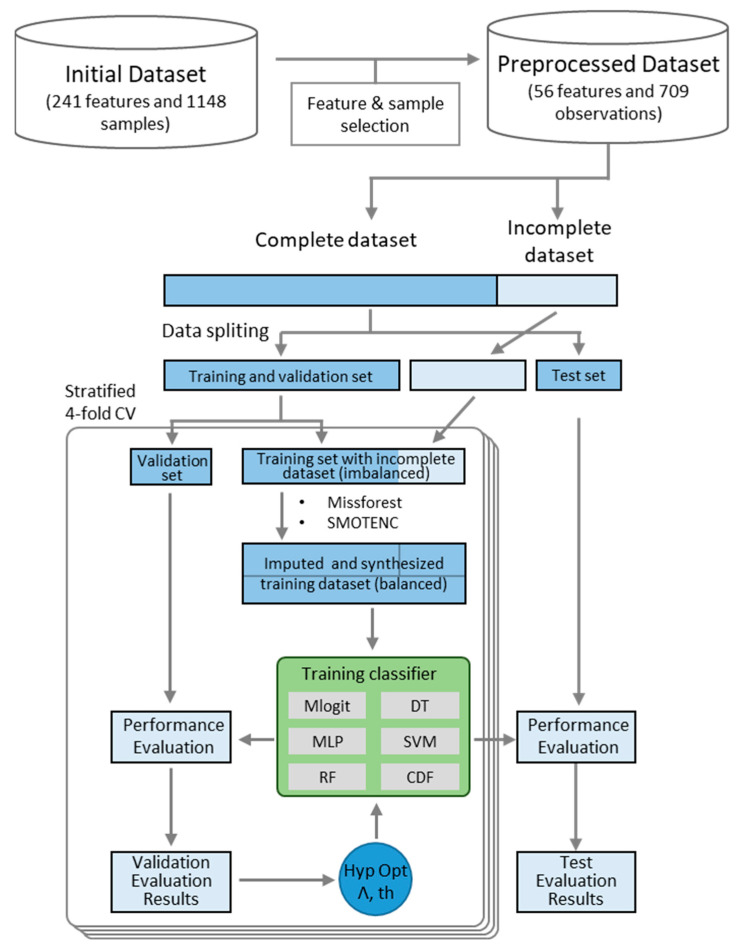
The overall process of developing an interpretable system for recommending herbal formulas.

In this study, we developed an HF recommendation model based on the clinical symptoms of patients (Figure 1). To improve its applicability and interpretability, we conducted data imputation on the training dataset using missForest and derived major features for the prediction results using LIME. Our model was developed using a multicenter study dataset collected by the Korean Institute of Oriental Medicine. We tried to construct a recommendation model with the best performance by comparing various classifiers, applying data imputation, and performing oversampling. The generalization ability of the developed model for predicting HFs was evaluated on the holdout test set and case study dataset. We also employed LIME to investigate the clinical symptoms that contributed to the predictions of administered HFs and evaluated their reliability and consistency. We believe that our model provides understandable recommendations for KM doctors who need decision making assistance and researchers who wish to study the HF selection process.

## 2. Materials and Methods

### 2.1. Data Collection and Selection Procedure

A dataset was obtained from the Korean Medicine Data Center (KDC) of the Korea Institute of Oriental Medicine. They conducted a clinical observational study between 2013 and 2015 with 10 Korean medicine clinics. One of the characteristics of the dataset is that it contains Sasang constitutional (SC) types diagnosed by licensed SCM specialists. Briefly, SC type is a typological concept used in SCM. In SCM, people are classified into four SC types, which are referred as Soeumin (SE), Soyangin (SY), Taeeumin (TE), and Taeyangin (TY) [14]. By using the diagnostic information of a patient’s SC type, an SCM expert can identify the risk factors for certain SC type-specific symptoms and provide a patient with tailored treatments. In this dataset, the diagnosis of the Sasang type was conducted by licensed SCM specialists who had been in clinical practice for at least 5 years. The SCM specialists diagnosed the Sasang type by carefully considering the physical body shapes, appearances, temperaments, and pathological symptoms of patients based on the detailed SC type determination processes that have been previously described [15]. All processes were approved by the Korea Institute of Oriental Medicine (I-1210/002-002-03), and written informed consent for participation was obtained from each subject.

The dataset initially included 241 measured clinical symptoms (features) and the resulting prescriptions (labels) for 1148 patients. We found that data selection was needed because some features were not closely related to clinical practice and some samples included sparsely appearing HFs. To this end, we selected the clinical symptoms and SC type, which were recorded as indicator symptoms in the clinical guidelines, as input characteristics. We also selected patients who received HFs that appeared at least 15 times in the dataset. For convenience, we refer to the constructed dataset as the KDC dataset.

### 2.2. Classifier Models

Supervised learning was conducted using various classifier models on the KDC dataset. Six classifiers were considered as potential classifiers for recommending HFs: logistic regression (logit), a decision tree (DT), a multilayer perceptron (MLP), a support vector machine (SVM), a random forest (RF), and a cascaded deep forest (CDF). These models showed sufficient performance in the recommendation system, and the CDF model was additionally considered as it yielded excellent performance in classification tasks including image classification. Logit is a type of linear regression method that adds a sigmoid function layer to the result of a linear mapping function. A DT uses a tree-like graph or models to make decisions and examine their possible outcomes. An MLP is a supervised learning algorithm that can learn nonlinear models. An SVM classifies outputs by finding the hyperplane that maximizes the margins between classes. An RF is an ensemble model that combines the probabilistic predictions of a number of DT-based classifiers to improve its generalization capability over that of a single estimator. A CDF employs a cascade structure, where the model consists of a multilayered architecture, and each level consists of an RF and extra trees [16]. The greedy search method was used to select the optimal hyperparameters of the classifiers within predetermined search ranges (Supplementary Table S1). Other hyperparameter not included in the search range were set to default values. CDF model and other classifier models was trained using DF21 module and scikit-learn module, respectively [16,17].

### 2.3. Data Oversampling

An imbalanced situation can be defined as a case in which the number of instances of the majority class is much higher than the number of minority class instances, and this problem can often be seen in multiclass classification. Having balanced data is important since machine learning algorithms may be biased toward the majority class. Oversampling is an efficient way to address this potential issue. One widely employed method for generating synthetic instances is the synthetic minority oversampling technique for nominal and continuous cases (SMOTE-NC) [18,19]. It is a variant of SMOTE and is suitable for use with datasets containing both continuous and categorical variables. SMOTE-NC uses the characteristics of the k-nearest neighbors classifier in the given feature space to generate synthetic data for the minority classes. It can effectively handle both numerical and categorical features that appear in the dataset. SMOTE-NC is conducted via the following procedure. (1) Take the training set obtained from the data splitting stage; (2) calculate the number of samples needed for resampling based on the number of samples of the majority class; (3) generate synthetic samples for the three minority classes (no damage, penetration, and scabbing) using SMOTE-NC; and (4) output the rebalanced dataset for training.

### 2.4. Data Imputation

MissForest is an RF-based data imputation method that can yield increased performance in classification problems relative to that of other methods when applied to a dataset containing both continuous and categorical variables [20]. Let the data matrix $X=\left(X_{1},\;X_{2},\;\ldots ,\;X_{p}\right)$ be an n-by-p matrix containing missing values, where n and p represent the numbers of subjects and features, respectively. Depending on the missing values, X is divided into four parts as follows: (1) the observed part of variable $X_{i}$ is denoted as $y_{obs}^{\left(s\right)}$; (2) the missing part of variable $X_{i}$ is denoted by $y_{mis}^{\left(s\right)}$; (3) variables other than $X_{i}$ with observation $i_{mis}^{\left(s\right)}$ are denoted by $x_{mis}^{\left(s\right)}$; and (4) variables other than $X_{i}$ with observation $i_{obs}^{\left(s\right)}$/$i_{mis}^{\left(s\right)}$ are denoted by $x_{obs}^{\left(s\right)}$. The missForest algorithm can be conducted as follows. (1) For a variable with missing data X, the missing values are replaced by the mean or mode of the remaining data; the mean is used for continuous variables, while the mode is employed for categorical variables. (2) For each variable with missing values, the $X_{i}$ built from the RF model on the observed $y_{obs}^{\left(s\right)}$ and $x_{obs}^{\left(s\right)}$ is grown, and the missing part $y_{mis}^{\left(s\right)}$ is predicted and replaced; this also based on the RF model. These processes are repeated until a stopping criterion is met or the maximum number of iterations is reached.

### 2.5. Case Study Selection and Data Processing

The selection of the case study, data curation approach, and data processing strategy were carried out according to the following procedure. We reviewed manuscripts published over 10 years (from July 2013 to June 2022) in the official journal of SCM (Journal of Sasang Constitutional Medicine) and initially collected 41 case studies and their full texts. The identifier of the case study was determined by the year-volume-issue-number of the published paper (i.e., 2018-30-4-06). The first author (W.Y. Lee) and independent curator carefully read each full manuscript and extracted the text concerning clinical symptoms and administered HFs into a structured table. The two authors (W.Y. Lee and J.H. Kim) read the extracted texts and discussed what values the clinical symptoms belonged to. Conflicts between the two authors over the assignment of values were resolved through discussions with other authors. During data curation, the authors found that some of the manuscripts in the case study did not fully describe the features defined in the KDC dataset and inevitably contained nan values (Supplementary Table S2). To conduct a performance evaluation, we selected 23 case studies in which more than half of the clinical symptoms were recorded and imputed the remaining clinical symptoms by using the missForest model trained on the KDC dataset.

### 2.6. LIME

LIME is essentially a model-agnostic interpretable framework that is utilized to explain the independent instance predictions of ‘black box’ machine learning models [13]. LIME conducts tests on what would happen to the predictions of a model when the user provides altered versions of their data instances to the model. LIME modifies a single data sample by tweaking its feature values and observes the resulting impact on the output. Based on this principle, LIME engenders a novel dataset comprising permuted samples and their corresponding predictions from the black box model. LIME was implemented in this study using the Python lime module (https://lime-ml.readthedocs.io/en/latest/, accessed on 3 March 2022).

### 2.7. Performance Evaluation

The performance achieved on the training set was evaluated using k-fold stratified cross-validation (CV). K-fold CV was conducted by dividing the training dataset into 5 folds, evaluating the performance on each fold individually, and utilizing the remaining folds for the model training. Stratified CV steps were used to adjust each CV fold to have the same proportion of administered HFs. For each fold of the predictive model, the following metrics were calculated:

Accuracy = (*TP* + *TN*)/(*TP* + *FP* + *FN* + *TN*)

Precision = *TP*/(*TP* + *FP*)

Recall = *TP/(TP* + *FN)*

F1 score = 2 * (Precision) * (Recall)/(Precision + Recall)

$$\mathrm{MCC}=(TP\times TN-FP\times FN)/\sqrt{\left(TP+FP\right)\left(TP+FN\right)\left(TN+FP\right)\left(TN+FN\right)},$$

where TP denotes true positives, FP represents false positives, FN denotes false negatives, and TN represents true negatives. To reduce the model variance, the performances of the classifier model were measured by conducting k-fold stratified CV several times. On the training dataset, the performance of the classifiers was evaluated under a condition where 5-fold CV was repeatedly conducted 5 times under different data splits. On the unseen dataset, the performance of the classifier was measured 10 times.

## 3. Results

### 3.1. Dataset Construction and Description

We obtained a dataset for developing a prescription recommendation model from the Korean Medicine Data Center of the Korea Institute of Oriental Medicine, hereafter referred to as the KDC dataset. By applying the selection criteria defined in the Methods section, we obtained a dataset for 973 patients with 54 clinical symptoms, SC types (features) and administered herbal prescriptions (labels). The included features for recommending HFs consisted of information on thirst, stool, urine, temperature and sweat, which are known clinical diagnostic indicators that are primarily relevant to prescription selection in KM. The name, type, description, scale, and distribution for the included features can be found in Supplementary Tables S3 and S4. We additionally considered the SC type as an input function, as this factor is known as useful information that can identify risk factors and provide customized treatments. The dataset also contained a total of 15 types of HFs, including 7 to 12 herbal medicines (Table 1).

We carefully examined the dataset and found two potential problems. First, we found significant deviations between the administered HM frequencies. For example, the top five prescriptions accounted for more than half of the total prescriptions (574/973). This type of imbalance can lead to shortened learning, where the model is trained with a bias toward labels that appear frequently in the training dataset. Second, our dataset contained samples for which the clinical symptoms were not fully recorded. A classifier trained with a model using only fully recorded features may have suboptimal performance. To address these potential issues, we tried to develop an optimal model that recommends HM through the following processes (Figure 1). First, we explored various classifiers and their hyperparameters using a fully valid dataset and selected the combination of classifiers and hyperparameters with the best predictive performance. Then, we evaluated whether the prediction performance was improved through the dataset obtained using oversampling and data imputation.

### 3.2. Hyperparameter Selection for the Classifiers

We first tried to select the best hyperparameters for each classifier model based on the KDC dataset. We first obtained features and their labels by selecting information from the 709 patients whose features were all validly recorded in the constructed dataset. The following classifiers were considered as potential models for predicting administered HFs: logit, DT, SVM, RF, MLP, and CDF models. A greedy search algorithm was used to find the optimized hyperparameters by comparing the performance of the classifier models in a predefined search range. We selected the optimized hyperparameters of the classifier model with the highest performance (Table 2). The result showed that the SVM, CDF, and MLP models achieved superior performance in terms of the top-1 accuracy, top-3 accuracy, and F1 score, respectively. Specifically, all classifiers achieved MCC values higher than zero, which indicates that a significant association is present between clinical symptoms and administered HMs.

### 3.3. Impact of Oversampling and Data Imputation

**Figure 2:**
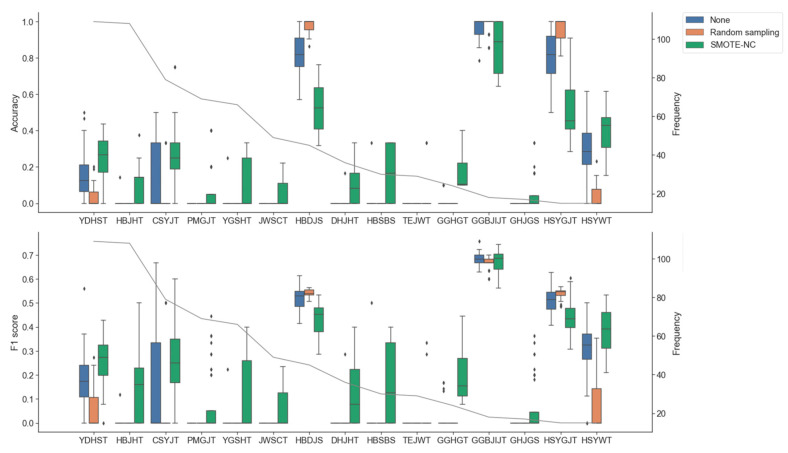
The distribution of F1 score and top-1 accuracy according to the oversampling method using cascade deep forest. The box color indicates the oversampling methods and the gray line refers to the frequency of herbal formulae shown in the KDC data set.

The above experiment did not adjust for data imbalance and excluded all samples containing nan values. This indicates that the prediction performance could be further improved by converting the dataset into a balanced dataset with imputed nan values. To this end, we first evaluated whether oversampling could improve the performance of the classifier models. We measured the prediction performance by conducting oversampling on the training set during the CV experiments (Table 2). Oversampling was performed by synthesizing samples belonging to the minority classes using random sampling and SMOTE-NC until the numbers of majority class and minority class samples matched. We found that using random sampling significantly lowered the top-3 accuracies, Macro-F1 and Macro-MCC values, indicating that random sampling may introduce overfitting issues. On the other hand, we found a significant improvement in the F1 scores and MCC values of all classifier models when utilizing SMOTE-NC. In particular, the CDF model improved its F1 score and MCC value substantially and achieved the highest top-1 accuracy, top-3 accuracy, and macro precision values. To understand the effect of oversampling, we further measure the predictive performance for each HM of the CDF model across oversampling methods. Without oversampling, we found that the accuracies and F1 scores of CDF models were highly biased by prescriptions (Figure 2 and Supplementary Table S5). This result indicates that accurate prediction can be performed only for a specific prescription. Introducing random sampling also did not improve or worsen the bias of performance. On the other hand, we found that the performance bias across HFs was significantly reduced when using SMOTE-NC. Specifically, applying SMOTE-NC achieved the best accuracy and F1 score for 80% of HFs (12/15). These results indicate that SMOTE-NC could be an efficient way to improve the performance of predicting various HFs.

**Figure 3:**
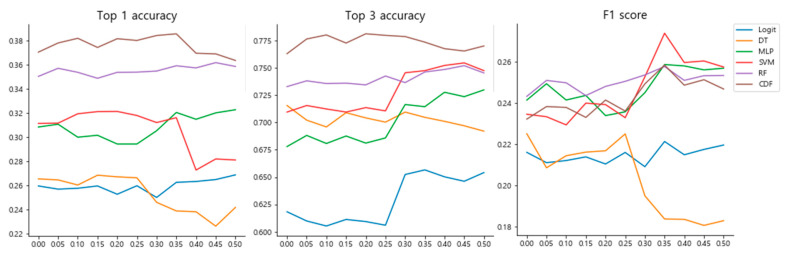
Performances of the classifier model with data imputation across the nan ratio threshold for the sample. Top-1 accuracy, top-3 accuracy, and F1 score of various classifiers were measured across various thresholds of nan ratios. Before performing classifier training, the nan values of the samples were imputed by the missForest model trained with valid samples.

We further evaluated whether data imputation could further improve performance. We expected that the performance of the recommendation model could be further improved if a sample with a high ratio of valid features acquired through imputation could be used for classifier training. We measured the prediction performance using a synthesized dataset by applying data imputation and oversampling to the training dataset. To find the optimal criteria for imputation, we selected the samples to be imputed and compared their performance while sequentially increasing the threshold of the nan ratio from 0 to 0.5. We found that the performance of classifier models progressively improved up to a nan ratio of 0.35 (Figure 3). In particular, the CDF model achieved a superior F1 score with the highest top-1 accuracy and top-3 accuracy at a threshold of 0.35. However, after this threshold, we found that the prediction performance decreased, especially for the F1 score, even if the nan ratio was higher. This result indicates that the imputation of appropriate samples and their utilization for subsequent supervised learning can contribute to constructing an optimized prediction model.

### 3.4. Evaluating the Model Generalization Abilities on Unseen Datasets

To test the generalization abilities of the models, the performance of the classifier models was further evaluated on unseen datasets. We first attempted to evaluate the performance of the trained classifier models on the holdout test set of the KDC dataset. We started by constructing a balanced dataset using data imputation and oversampling on the training set of the KDC dataset. We trained the classifier models on this dataset and predict the labels (administered HFs) using the features of the holdout test set. The results showed that the trained models achieved superior performance even on unseen datasets (Table 3). Specifically, we found that the CDF model still had the highest top-3 accuracy and high F1 scores. This result indicates that the predictions of our model did not have a significant overfitting issue.

To evaluate the practical applicability of our model, we constructed a case study dataset and used it for evaluation. We expected that if our model correctly estimated the actual administered HFs using recorded clinical symptoms, then it could be usefully used in the real world as well. Through the selection criteria and data processing strategy defined in the Methods section, we obtained the clinical symptoms and administered HFs for 23 published case studies. We predicted the HFs of the case study using a classifier model trained on the dataset obtained by applying data imputation and oversampling to the entire KDC dataset. We found that our classifier model performed much better than it did on the training set (Table 3). In particular, we found that the CDF model still had the highest top-3 accuracy and F1 score. It is noteworthy that the proportion of valid features in the case study was only 0.5–0.68, indicating that the classifier model accurately predicted HFs even with data in which only partially recorded features were recorded.

### 3.5. Model Interpretation Using Case Study Data

Understanding what a trained classifier learns in a data-driven manner may provide valuable clinical insights. Therefore, we applied an explainability technique called LIME to highlight which clinical symptoms drove particular HFs. The CDF model was selected as the classifier model to which LIME was applied, as it achieved superior performance across various experiments. We selected a case study (case id: 2018-30-4-06) and applied LIME as a representative example [21]. This case study concerned a patient who visited the KM hospital with an essential tremor after undergoing a total vaginal hysterectomy. After considering the clinical symptoms, the authors of the case study administered HBJHT, which was consistent with our model’s predictions. We computed the weights of the features that contributed to the prediction using LIME and represented the top 10 features and their descriptions (Table 4). The weights of the features derived from LIME can be understood to the extent that the features affected the HF selection outcome. For example, the increase in the feature values of Stool_form, Fatigue and Stool_tenesmus would have contributed more positively to the HBJHT administration probability provided by the recommender system. On the other hand, the decreases in Stool_frequency and stress would have contributed more positively to the probabilities of the administered HFs. These results indicate that the application of LIME aids in producing a more reliable recommendation system by providing clinicians with criteria for evaluating prescription choices.

**Figure 4:**
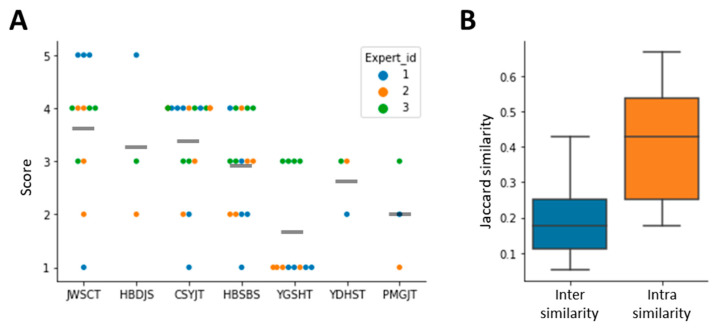
Evaluation score and consistency of major features derived by applying LIME. (**A**) Score distribution of LIME analysis results evaluated by experts. The gray bar represents the average value of the score for each HF. (**B**) Similarity between major features identified by LIME.

We tried to evaluate the interpretation results by expert’s manual audit. The remaining 22 instances included in the case study dataset were considered as data for model evaluation. We applied LIME to each instance consisting of the patient’s clinical symptoms and administered HFs, and identified the top-weighted features and their descriptions in a form similar to the representative example. We then invited three SCM experts to rate whether these features were appropriate for choosing a HFs on a scale of 1 (most inappropriate) to 5 (most appropriate). The results showed that the average score of evaluation was close to normal (2.94) (Figure 4A). JWSCT had the highest average score with 3.7 points, followed by CSYJT (3.4 points) and HBDJS (3.3 points). Then, we evaluate the consistency of the interpretation results. We expected that if the overlap between the features with high weights in the same prescription was higher than that between other features, it would support the consistency of our model. We calculated the weight of each clinical symptom for the top predicted HF from each case study dataset using LIME. Then, we calculated the Jaccard similarities between the top 10 features of the same prescription (intra-similarity) and different prescriptions (inter-similarity). The results showed that the intrasimilarity was significantly higher than the intersimilarity (*p* < 0.001, Figure 4B), indicating that the LIME model interprets with consistent criteria.

## 4. Discussion

KM has been a practical science for thousands of years, and KM doctors have long been prescribing HFs based on the clinical symptoms of patients. Experiences on the efficacy of using HFs and herbal medicines have been accumulated, but they are mainly implicit and cannot be quantitatively measured. In this study, we proposed an HF recommendation model with enhanced applicability and interpretability. To our knowledge, this is the first approach that can explicitly identify the HF recommendation process. In addition, our model enhanced its practicality and usefulness by using only the clinical information defined in the clinical treatment guidelines as input data for HF recommendation. We also proposed a comprehensive strategy that combines various classifiers, data imputation, and oversampling to develop models with optimal predictive performance. A comprehensive evaluation demonstrated that our model accurately predicts HFs, even on the holdout dataset and a partially recorded case study dataset. The applied LIME model was able to identify the clinical symptoms that were mainly considered in the HF recommendation process.

We proposed an HF recommendation model using a comparison between classifiers, a data imputation strategy, and oversampling techniques. Among the classifier models, we found that the RF and CDF model achieved the highest performance in unseen datasets. Random forest requires less data preparation and preprocessing than neural networks or SVMs and is known to exhibit superior performance on various datasets. Because of this usefulness, random forests have been successfully applied in recommendation systems in the biomedical field [22,23]. The CDF model, a random forest-based ensemble model, automatically determined the appropriate complexity in a data-dependent way with relatively few parameters and achieved excellent performance across various domains. Additionally, we confirmed that data imputation and oversampling can improve the performance of the recommendation system. These results show that an appropriate data preprocessing approach is an efficient way to solve incomplete and imbalanced data issues, which are unavoidable for most multiclassification problems in the biomedical field. Finally, the robust performance of our recommendation system was evaluated on various holdout test sets and a case study dataset. In particular, the superior performance achieved on the case study dataset with partially recorded features supported the practical applicability of the developed system. Taken together, we suggest that combining various classifiers, data imputation methods, and oversampling techniques can be an efficient strategy for developing machine learning models with optimal performance and high applicability.

This study exhibits several limitations with the potential for further improvement. First, our recommendation model is limited to 15 types of HF recommendations. If a dataset consisting of various HFs is secured, our model can be extended to recommend various HFs. Second, the prediction performance can be further improved by applying advanced algorithms, such as graph convolutional networks (GCNs), which have been recently reported to achieve state-of-the-art performance [12]. In addition, expert evaluation results suggested that there is room for improvement in the interpretation results of our model. Finally, although our model only requires information used in the clinical setting, but it requires the additional process of recording clinical information in a predefined format to recommend HFs. An interesting future research topic is to improve the explainability of models by combining state-of-the-art models such as DLP or to develop models based on natural language process models using directly progress notes recorded in clinical practice as input data [24]. Despite these limitations, we are the first to propose an interpretable HF recommendation model, which will contribute to the development of explicit knowledge for recommending HFs and the modernization of KM.

## Figures and Tables

**Table 1:** Names of the herbal formulas included in the recommendation system and their compositions.

Formula Name (Abbreviation)	Composition (per Serving)
Cheongsimyeonja-tang (CSYJT)	*Nelumbinis Semen*, *Dioscoreae Rhizoma*, *Asparagi Tuber*, *Polygalae Radix*, *Acori Graminei Rhizoma*, *Zizyphi Semen*, *Longan Arillus*, *Thujae Semen*, *Scutellariae Radix*, *and Raphani Semen* 7.5 g, *Chrysanthemi Flos* 1.125 g
Dokhwaljihwang-tang (DHJHT)	*Rehmanniae Radix Preparata* 15 g, *Corni Fructus* 7.5 g, *Poria Sclerotium*, *and Alismatis Rhizoma* 5.625 g, *Moutan Cortex*, *Saposhnikoviae Radix*, *and Angelicae Continentalis Radix* 3.75 g
Galgeunhaegi-tang (GGHGT)	*Puerariae Radix* 11.25 g, *Cimicifugae Rhizoma* 7.5 g, *Scutellariae Radix*, *and Armeniacae Semen* 5.625 g, *Platycodonis Radix*, *Zizyphi Semen*, *Rhei Rhizoma*, *and Angelicae Dahuricae Radix* 3.75 g
Gwakhyangjeonggi-san (GHJGS)	*Agastachis Herba* 5.625 g, *Perillae Folium* 3.75 g, *Attactylodis Rhizoma*, *Attactylodis Rhizoma Alba*, *Pinelliae Tuber*, *Citri Unshius Pericarpium*, *Arecae Pericarpium*, *Citri Unshius Pericarpium Immaturus*, *Cinnamomi Cortex*, *Zingiberis Rhizoma*, *Alpiniae Oxyphyllae Fructus*, *and Glycyrrhizae Radix et Rhizoma* 1.875 g
Gwangyebujaijung-tang (GGBJIJT)	*Ginseng Radix* 11.25 g, *Attactylodis Rhizoma Alba*, *Zingiberis Rhizoma* (stir-bake), *and Cinnamomi Cortex* 7.5 g, *Paeoniae Radix Alba*, *Citri Unshius Pericarpium*, *and Glycyrrhizae Radix et Rhizoma* (stir-bake) 3.75 g, *Aconiti Lateralis Radix Preparata* 3.75 or 7.5 g
Hyangbujapalmul-tang (HBJPMT)	*Cyperi Rhizoma*, *Attractylodis Rhizoma Alba*, *Poria Sclerotium* (white), *Pinelliae Tuber*, *Citri Unshius Pericarpium*, *Magnoliae Cortex*, *and Amomi Fructus Rotundus* 3.75 g, *Ginseng Radix*, *Glycyrrhizae Radix et Rhizoma*, *Aucklandiae Radix*, *Amomi Fructus*, *and Alpiniae Oxyphyllae Fructus* 1.875 g, *Zingiberis Rhizoma Crudus* 3 slices, *Zizyphi Fructus* 2 pulps
Hyangsayangwi-tang (HSYWT)	*Ginseng Radix*, *Attactylodis Rhizoma Alba*, *Paeoniae Radix Alba*, *Glycyrrhizae Radix et Rhizoma (stir-bake)*, *Cyperi Rhizoma*, *Citri Unshius Pericarpium*, *Zingiberis Rhizoma*, *Crataegi Fructus*, *Amoni Fructus*, *and Amomi Fructus Rotundus* 3.75 g
Hyeongbangdojeok-san (HBDJS)	*Rehmanniae Radix Crudus* 11.25 g, *Akebiae Caulis* 7.5 g, *Scrophulariae Radix*, *Trichosanthis Semen*, *Angelicae Decursivae Radix*, *Osterici Radix*, *Angelicae Continentalis Radix*, *Schizonepetae Spica*, *and Saposhnikoviae Radix* 3.75 g
Hyeongbangjihwang-tang (HBJHT)	*Rehmanniae Radix Preparata*, *Corni Fructus*, *Poria Sclerotium*, *and Alismatis Rhizoma* 7.5 g, *Plantaginis Semen*, *Osterici Radix*, *Angelicae Continetalis Radix*, *Schzonepetae Spca*, *and Saposhnikoviae Radix* 3.75 g
Hyeongbangsabak-san (HBSBS)	*Rehmanniae Radix Crudus* 11.25 g, *Poria Sclerotium* 7.5 g, *Gypsum Fibrosum*, *Anemarrhenae Rhizoma*, *Osterici Radix*, *Schizonepetae Spica*, *and Saposhnikoviae Radix* 3.75 g
Jowiseungcheong-tang (JWSCT)	*Coisis Semen*, *and Castaneae Semen* 11.25 g, *Raphani Semene* 5.625 g, *Ephedrae Herba*, *Platycodonis Radix*, *Liriopis Tuber*, *Schisandrae Fructure*, *Acori Graminei Rhizome*, *Polygalae Radix*, *Asparagi Tuber*, *Zizyphi semen*, *and Longan Arillus* 3.75 g
Palmulgunja-tang (PMGJT)	*Ginseng Radix* 7.5 g, *Astragali Radix*, *Attactylodis Thizoma Alba*, *Paeoniae Radix Alba*, *Angelicae Gigantis Radix*, *Cnidii Rhizoma*, *Citri Unshius Pericarpium*, *and Glycyrrhizae Radix et Rhizoma (stir-bake)* 3.75 g, *Zizyphi Fructurs* 2 pulps
Taeeumjowi-tang (TEJWT)	*Coisis Semen*, *and Castaneae Semen* 11.25 g, *Raphani Semen* 7.5 g, *Schisandrae Fructus*, *Liriopis Tuber*, *Acori Graminei Rhizoma*, *Platycodonis Radix*, *and Ephedrae Herba* 3.75 g
Yanggyeoksanhwa-tang (YGSHT)	*Rehmanniae Radix Crudus*, *Lonicerae Folium et Caulis*, *and Forsythiae Fructus* 7.5 g, *Gardeniae Fructus*, *Menthae Herba*, *Anemarrhenae Rhizoma*, *Gypsum Fibrosum*, *Saposhinikoviae Radix*, *and Schizonepetae Spica* 3.75 g
Yeoldahanso-tang (YDHST)	*Puerariae Radix* 15 g, *Scutellariae Radix*, *and Angelicae Tenuissimae Radix* 7.5 g, *Raphani Semen*, *Platycodonis Radix*, *Cimicifugae Rhizoma*, *and Angelicae Dahuricae Radix* 3.75 g

**Table 2:** Performance distribution on the KDC dataset across classifier models by oversampling methods.

Oversampling	Classifier Models	Top-1 Accuracy	Top-3 Accuracy	Macro-F1	Macro-MCC ^#^
None	Logit	0.365 ± 0.003	0.674 ± 0.002	0.151 ± 0.002	0.116 ± 0.002
	DT	0.347 ± 0.006	0.725 ± 0.004	0.222 ± 0.005	**0.211 ± 0.007**
	MLP	0.334 ± 0.005	0.691 ± 0.004	**0.242 ± 0.006**	0.198 ± 0.005
	SVM	**0.389 ± 0.003**	0.739 ± 0.002	0.237 ± 0.003	0.168 ± 0.006
	RF	0.388 ± 0.005	0.767 ± 0.003	0.193 ± 0.006	0.165 ± 0.006
	CDF	0.380 ± 0.004	**0.786 ± 0.003**	0.165 ± 0.003	0.140 ± 0.005
Random	Logit	0.235 ± 0.002	0.589 ± 0.003	0.203 ± 0.003	0.161 ± 0.004
Sampling	DT	0.194 ± 0.006	0.693 ± 0.006	0.153 ± 0.005	0.120 ± 0.005
	MLP	0.310 ± 0.005	0.668 ± 0.006	**0.235 ± 0.004**	**0.191 ± 0.004**
	SVM	0.304 ± 0.005	0.716 ± 0.003	0.208 ± 0.005	0.162 ± 0.005
	RF	0.357 ± 0.004	0.753 ± 0.005	0.221 ± 0.007	0.187 ± 0.008
	CDF	**0.400 ± 0.002**	**0.754 ± 0.002**	0.131 ± 0.002	0.125 ± 0.002
SMOTENC	Logit	0.253 ± 0.003	0.617 ± 0.005	0.211 ± 0.004	0.167 ± 0.006
	DT	0.265 ± 0.006	0.714 ± 0.005	0.220 ± 0.005	0.178 ± 0.008
	MLP	0.312 ± 0.005	0.683 ± 0.005	**0.246 ± 0.005**	0.195 ± 0.005
	SVM	0.310 ± 0.004	0.716 ± 0.004	0.237 ± 0.004	0.176 ± 0.006
	RF	0.349 ± 0.005	0.728 ± 0.006	0.238 ± 0.007	**0.196 ± 0.010**
	CDF	**0.376 ± 0.006**	**0.773 ± 0.006**	0.232 ± 0.008	0.189 ± 0.008

Data are expressed as mean ± standard error. ^#^ MCC; Matthew’s correlation coefficient.

**Table 3:** Performance evaluation results obtained on various unseen datasets.

Datasets	Classifier Models	Top-1 Accuracy	Top-3 Accuracy	Macro-F1
Holdout	Logit	0.247 ± 0.005	0.660 ± 0.005	0.227 ± 0.006
test set	DT	0.242 ± 0.013	0.698 ± 0.008	0.189 ± 0.012
	MLP	0.326 ± 0.009	0.725 ± 0.012	0.270 ± 0.010
	SVM	0.263 ± 0.007	0.752 ± 0.008	0.249 ± 0.004
	RF	**0.349 ± 0.008**	0.766 ± 0.005	**0.257 ± 0.007**
	CDF	0.342 ± 0.005	**0.785 ± 0.006**	0.247 ± 0.008
Case study	Logit	0.339 ± 0.023	0.835 ± 0.016	0.233 ± 0.027
dataset	DT	0.270 ± 0.037	0.765 ± 0.015	0.183 ± 0.028
	MLP	0.291 ± 0.031	0.830 ± 0.019	0.278 ± 0.022
	SVM	0.287 ± 0.019	0.835 ± 0.017	0.202 ± 0.011
	RF	**0.361 ± 0.022**	0.865 ± 0.016	0.283 ± 0.017
	CDF	0.352 ± 0.031	**0.896 ± 0.013**	**0.298 ± 0.025**

**Table 4:** A representative example of the analysis results obtained by applying LIME.

Feature Class	Weight	Feature Value	Feature Description
SC type	0.39	3 (So-Yang type)	1: Tae-yang type 2: So-eum type 3: So-yang type 4: Tae-yang type
Stool_frequency *	−0.02	2	(frequency per day)
Stool_frequency †	−0.01	2	(frequency per day)
Stool_form *	0.01	4 (Mushy consistency with ragged edges)	1: Separate hard lumps, 2: A sausage shape with cracks in the surface, 3: Soft blobs with clear-cut edges, 4: Mushy consistency with ragged edges, 5: Watery diarrhea
Stress †	−0.01	3 (Moderate)	1: None, 2: Mild, 3: Moderate, 4: Severe, 5: Very severe
Fatigue *	0.01	4 (Severe)	1: None, 2: Mild, 3: Moderate, 4: Severe, 5: Very severe
Stool_tenesmus *	0.01	3 (Moderate)	1: None, 2: Mild, 3: Moderate, 4: Severe, 5: Very severe
Complexion_red †	0.01	3 (Moderate)	1: None, 2: Mild, 3: Moderate, 4: Severe, 5: Very severe
Digestion †	0.01	4 (Poor)	1: Very good, 2: Good, 3: Fair, 4: Poor, 5: Very poor
Stool_form†	0.01	4 (Mushy consistency with ragged edges)	1: Separate hard lumps, 2: A sausage shape with cracks in the surface, 3: Soft blobs with clear-cut edges, 4: Mushy consistency with ragged edges, 5: Watery diarrhea

*: Original symptom, †: present symptom.

## Data Availability

The data supporting the findings of this study are available from the Korea medicine Data Center (KDC). Data can be made available from the authors upon reasonable request and with permission of the KDC and the Institutional Data Access/Ethics Committee of Korea Institute of Oriental Medicine. Requests for access to these data can be made at the KDC website (kdc.kiom.re.kr, accessed on 3 March 2022).

## Supplementary Materials

The following supporting information can be downloaded at https://www.mdpi.com/article/10.3390/biom12111604/s1, Supplementary Table S1: Search range and selected hyperparameter values for the classifier models. Supplementary Table S2: The publication information and properties of case studies in Journal of Sasang Constitutional Medicine. Supplementary Table S3: Description of the input feature used in the recommendation model. Supplementary Table S4: Distribution of features according to the classes of Sasang constitutional type. Supplementary Table S5: The distribution of F1 score and top-1 accuracy according to the oversampling method using cascade deep forest.
